# Primary Tubercular Chorioretinitis Without a Pulmonary Focus: A Case Report

**DOI:** 10.7759/cureus.57905

**Published:** 2024-04-09

**Authors:** Tyfur Rahman, Uma Gupta, Urmi Das, Tanzima Afrin, Tahmina Akter

**Affiliations:** 1 Internal Medicine, Institute of Applied Health Sciences, Chittagong, BGD; 2 Internal Medicine, One Brooklyn Health/Interfaith Medical Center, New York, USA; 3 Internal Medicine, Chittagong Medical College and Hospital, Chittagong, BGD; 4 Medicine, Rajshahi Medical College, Rajshahi, BGD; 5 Medicine, North East Medical College and Hospital, Sylhet, BGD

**Keywords:** antitubercular therapy, medicine in resource-limited areas, tuberculin skin test, mantoux test, tuberculosis, fundoscopy, optical coherence tomography (oct), tubercle, ocular tuberculosis, tubercular chorioretinitis

## Abstract

Ocular tuberculosis (TB) can affect various eye structures and may manifest independently of systemic TB. Typically, it arises from hematogenous dissemination from a primary focus; however, in exceptional instances, it may originate as a primary infection after epithelial injury. Diagnosing TB in an extrapulmonary site presents a significant clinical challenge. We present the case of a 33-year-old Bangladeshi female who presented with a deteriorating loss of vision in her left eye. A thorough neurologic examination and serological tests, the tuberculin skin test, a CT scan of the chest, ocular fundus photography, and optical coherence tomography were performed. Based on the clinical features and the outcome of appropriate tests, a presumptive diagnosis of ocular TB was made and later confirmed after initiating antitubercular therapy, which resulted in a marked improvement in the patient’s vision a week later. This case is an illustration of the rare nature and unusual presentation of extrapulmonary TB in the form of tubercular chorioretinitis, diagnosed in a resource-limited setting. Tubercular chorioretinitis, characterized by inflammation of the choroid and retina due to TB infection, presents a diagnostic challenge, especially in resource-limited environments where access to advanced diagnostic tools may be restricted. Therefore, this case highlights the importance of considering TB as a potential cause of ocular manifestations, even in settings where TB prevalence might not be high, and underscores the need for increased awareness and diagnostic capacity for extrapulmonary TB in resource-limited areas. This case exemplifies the infrequent occurrence and atypical manifestation, presenting a learning opportunity for future clinicians.

## Introduction

Tuberculosis (TB) is a global health problem. Around 2 billion people, or a quarter of the world’s population, could have TB, with 10.6 million falling ill each year. TB remains deadly despite being preventable and treatable, claiming over 3,500 lives daily and totaling 1.3 million deaths annually. Additionally, approximately 30% of TB cases are missed by healthcare screenings and diagnostics, leading to poor health outcomes and further TB spread in communities. Untreated individuals can infect 10 to 15 others per year, with 10% developing active TB in their lifetimes [1]. TB is an infectious disease that can affect various organs in the body, including the eye. The infections inflicted by TB can occur with or without evidence of systemic TB. The infection can develop following hematogenous spread, but can also occur following direct epithelial injury. Ocular manifestations of TB can mimic those of other inflammatory diseases. Establishing the diagnosis of ocular TB is a clinical challenge, as most tubercular infections affect the pulmonary system first, and primary infection of the eye is extremely rare, with an incidence of about 1% to 2% [2]. The diagnosis of ocular TB warrants a combination of ocular inflammatory signs and the demonstration of mycobacterial culture or DNA amplification of the ocular samples. A presumptive diagnosis can be proved by a combination of ophthalmologic findings consistent with TB and tests that can confirm a TB infection, such as the interferon-gamma release assay (IGRA) or the tuberculin skin test (TST) [3]. Ocular TB remains undiagnosed due to the absence of ocular biopsies, and biopsy of the retina is generally not possible. We report a case of tubercular chorioretinitis, choroidal tubercles, a very rare finding of extrapulmonary TB, diagnosed based on clinical features, optical coherence tomography (OCT) imaging findings, and a positive TST in a highly endemic setting.

## Case presentation

A 33-year-old female with no previous history of diabetes or hypertension presented with blurred vision in the left eye associated with redness and pain for one month. She had a history of subjective, intermittent low-grade fever for three months, accompanied by anorexia and weight loss. While she lacked an evident contact history, she hailed from a region endemic to TB. Her immunization status was unknown. On examination of the respiratory system, there were no abnormalities. The examination of other systems was also unremarkable. Her ophthalmologic examination showed significantly decreased visual acuity in the left eye to light perception. The visual acuity in her right eye was 6/6. Regarding the field of vision, the direct and consensual light reflex was lost and absent in the left eye, which was dilated. Additionally, her accommodation was normal. On further examination, the vitreous was normal, with no signs of anterior segment inflammation. Both the intraocular pressure and anterior chamber angle were within normal limits. Initially, pupil dilation was observed as a medication-induced effect. Subsequent examinations revealed no abnormalities. An indirect funduscopic examination showed a hyperemic disc with blurred margins and an obliterated cup-to-disk ratio due to disc edema in the left eye. There were no abnormalities on the oculomotor examination.

**Figure 1:**
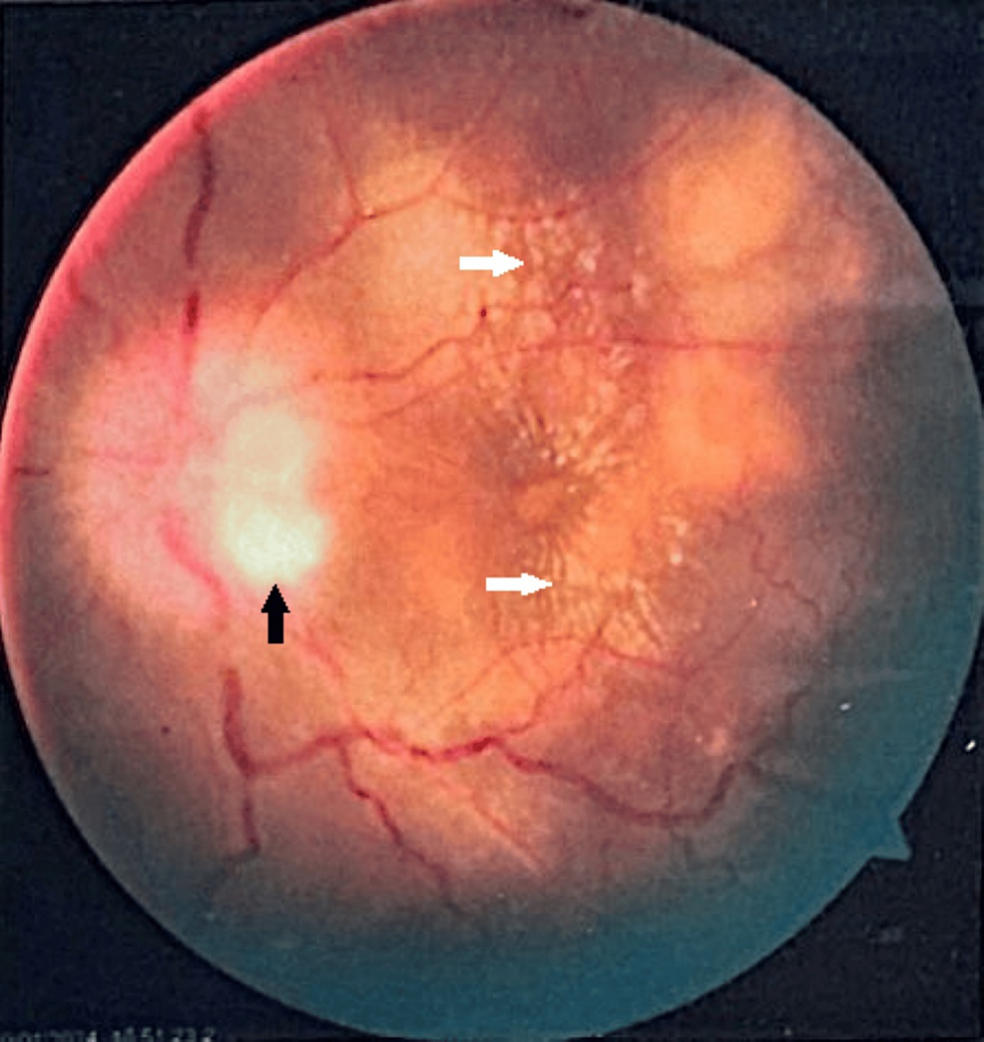
Fundus photograph showing papilledema and irregular optic cup-to-disk ratio (black arrow) with macular exudates (white arrows).

**Figure 2:**
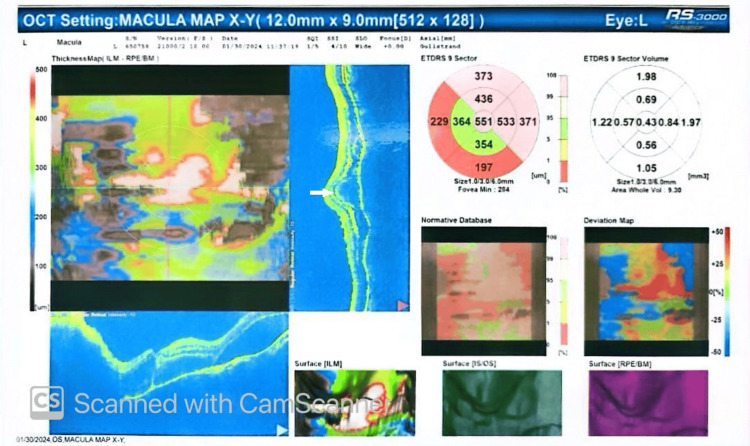
Optical coherence tomography showing an intraretinal lesion (white arrow).

Her complete blood count was significant for hemoglobin of 11.7 g/dL, erythrocyte sedimentation rate of 45 mm in the first hour, and C-reactive protein of 11.8 mg/dL. Her serum electrolytes, creatinine, hepatitis B surface antigen, hepatitis C virus antibody test, Venereal Disease Research Laboratory test, *Treponema pallidum* hemagglutination assay, and human immunodeficiency virus were unremarkable (Table 1). A CT scan of the chest showed no evidence of consolidation, collapse, or fibrosis. No hilar lymphadenopathy was observed, the cardiac shadow exhibited a normal outline, and the trachea was centrally positioned. A CT scan of the head showed no abnormalities. Her Mantoux test was positive with an induration of 14 mm (Table 2). Fundoscopy in the left eye showed papilledema with exudates around the macula (Figure 1). Her OCT showed a choroidal tubercle and a choroidal granulomatous lesion (Figure 2). Based on her demographic characteristics, clinical examination, positive TST, fundoscopy, and OCT findings, she was diagnosed with ocular TB. She was initiated on standard antitubercular therapy (ATT): rifampicin 150 mg, isoniazid 75 mg, pyrazinamide 400 mg, ethambutol HCL 275 mg, along with systemic corticosteroids (30 mg PO daily).

**Table 1:** Serological tests with reference ranges. ESR = erythrocyte sedimentation rate; CRP = C-reactive protein; HIV = human immunodeficiency virus; HBsAg = hepatitis B surface antigen; anti-HCV = hepatitis C virus antibody; VDRL = Venereal Disease Research Laboratory; TPHA = *Treponema pallidum* hemagglutination assay

Test	Results	Reference value
Serum sodium	141 mmol/L	135–148 mmol/L
Serum potassium	4.3 mmol/L	3.5–5.3 mmol/L
Serum chloride	103.0 mmol/L	98–107 mmol/L
Serum bicarbonate	21.0 mmol/L	22–29 mmol/L
Serum creatinine	0.48 mg/dL	0.6–1.4 mg/dL
Hemoglobin	11.7 g/dL	Female = 12–15 g/dL; male = 13–17 g/dL
ESR	45 mm	At the end of the first hour: male = 0–10 mm; female = 0–15 mm
CRP	11.8 mg/L	<3.0 mg/dL
Serological tests		
HIV	Negative	-
HBsAg	Negative	-
Anti-HCV	Negative	-
VDRL	Non-reactive	-
TPHA	Titer <1:80	-
	Opinion: negative	-

**Table 2:** Tuberculin skin test showing 14 mm induration.

Test	Result
Date of injection	January 30, 2024, 02:06 PM
Dose of injection	10 T.U. of PPD
Date of observation	February 2, 2024, 11:28 AM
On observation	Induration present
Diameter	14 mm
Interpretation	Positive

A week after her treatment, a notable enhancement in her visual acuity to 6/9 was observed, accompanied by reduced optic disc swelling, as evidenced by indirect fundoscopy. The ocular examination findings, along with documented imaging during the follow-up period, demonstrated a significant improvement in visual acuity. We assessed visual acuity and funduscopic findings as indicators of improvement. Subsequently, a referral was made to a vitreo-retina specialist for a three-month follow-up. A repeat IGRA yielded a positive result. She responded well to ATT with steroids, confirming the diagnosis of ocular TB.

## Discussion

Early diagnosis and treatment of ocular TB are vital to preserve vision and limit its spread. The documented occurrence of tubercular uveitis (TBU) shows considerable variation globally. It ranges from 0.2%-2.7% in regions where TB is not widespread, like the United States, Europe, or Japan, to 5.6%-10.5% in highly affected areas such as India [4,5]. We diagnosed our case in Bangladesh, a highly endemic region for TB. The demographic setting and densely populated area provided us with a high level of suspicion for extrapulmonary TB, which prompted further evaluation. We advised relevant investigations that allowed us to reach our presumptive diagnosis, and a positive TST made ocular TB the most probable diagnosis. The Collaborative Ocular Tuberculosis Study-1 group indicated that positive or negative test outcomes do not impact the treatment approach for the condition in real-world situations. This is because of the low sensitivity and absence of standardization [6]. Tubercular posterior uveitis (TPU), specifically tubercular choroiditis, is the most common form of TBU [7,8]. Tubercular serpiginous-like choroiditis appears as multiple, yellowish lesions with slightly raised edges that are not connected to the optic disc [8-11]. In the hematogenous spread of *Mycobacterium tuberculosis* (MTB), tubercles typically manifest as multiple, small, grayish-yellowish nodules. In our case, the patient presented with a choroidal tubercle and choroidal granulomatous lesion consistent with tuberculous granuloma associated with papilledema and macular exudates. The primary support for the direct involvement of MTB in the development of ocular TB lies in the positive impact of ATT on resolving inflammation or preventing its recurrence [12,13]. Conversely, the indirect influence of MTB is primarily backed by the absence of microbiological or molecular evidence of MTB in ocular fluid samples and the therapeutic effectiveness of corticosteroid therapy alone in clinically diagnosed ocular TB cases [12]. Similarly, in our case, the initiation of treatment with marked improvement of vision further confirmed the diagnosis of ocular TB in our patient.

## Conclusions

We highlight the importance of a comprehensive clinical approach to facilitate diagnosing ocular TB. Following ATT (treatment protocol), the patient improved which prevented irreversible loss of vision. Our report emphasizes the need for increased awareness to alleviate the impact of any debilitating outcome. Further research and follow-up are important to enhance our understanding and treatment of ocular TB globally.

## References

[1] (2024). Global TB overview. https://www.cdc.gov/globalhivtb/who-we-are/about-us/globaltb/globaltb.html.

[2] Donahue HC (1967). Ophthalmologic experience in a tuberculosis sanatorium. Am J Ophthalmol.

[3] Pirraglia MP, Tortorella P, Abbouda A, Toccaceli F, La Cava M (2015). Spectral domain optical coherence tomography imaging of tubercular chorioretinitis and intraretinal granuloma. Intraretinal tuberculosis: a case report. Int Ophthalmol.

[4] Ang M, Chee SP (2017). Controversies in ocular tuberculosis. Br J Ophthalmol.

[5] Abu El-Asrar AM, Abouammoh M, Al-Mezaine HS (2010). Tuberculous uveitis. Int Ophthalmol Clin.

[6] Agarwal A, Agrawal R, Gunasekaran DV (2019). The Collaborative Ocular Tuberculosis Study (COTS)-1 report 3: polymerase chain reaction in the diagnosis and management of tubercular uveitis: global trends. Ocul Immunol Inflamm.

[7] Gupta A, Sharma A, Bansal R, Sharma K (2015). Classification of intraocular tuberculosis. Ocul Immunol Inflamm.

[8] Agrawal R, Agarwal A, Jabs DA (2020). Standardization of nomenclature for ocular tuberculosis - results of Collaborative Ocular Tuberculosis Study (COTS) Workshop. Ocul Immunol Inflamm.

[9] Gupta V, Gupta A, Rao NA (2007). Intraocular tuberculosis--an update. Surv Ophthalmol.

[10] Nazari Khanamiri H, Rao NA (2013). Serpiginous choroiditis and infectious multifocal serpiginoid choroiditis. Surv Ophthalmol.

[11] Bansal R, Gupta A, Gupta V, Dogra MR, Sharma A, Bambery P (2012). Tubercular serpiginous-like choroiditis presenting as multifocal serpiginoid choroiditis. Ophthalmology.

[12] Agrawal R, Gunasekeran DV, Grant R (2017). Clinical features and outcomes of patients with tubercular uveitis treated with antitubercular therapy in the Collaborative Ocular Tuberculosis Study (COTS)-1. JAMA Ophthalmol.

[13] Kee AR, Gonzalez-Lopez JJ, Al-Hity A (2016). Anti-tubercular therapy for intraocular tuberculosis: a systematic review and meta-analysis. Surv Ophthalmol.

